# Risk Factors for Facial Pain: Data from the Osteoarthritis Initiative Study

**Published:** 2017-02-09

**Authors:** Qoot Alkhubaizi, John David Sorkin, Marc C Hochberg, Sharon M Gordon

**Affiliations:** 1 Department of General Dental Practice, Faculty of Dentistry, Kuwait University, Kuwait; 2 Geriatric Research, Education and Clinical Center, Baltimore VA Medical Center, Baltimore, USA; 3 Medical Care Clinical Center, VA Maryland Health Care System, Baltimore, USA; 4 Division of Gerontology and Geriatric Medicine, Department of Medicine, University of Maryland School of Medicine, Baltimore, USA; 5 Departments of Medicine and Epidemiology and Public Health, University of Maryland School of Medicine, Baltimore, USA; 6 Department of Foundational Sciences, School of Dental Medicine, East Carolina University, Greenville, USA

**Keywords:** Pain, Epidemiology, Temporomandibular disorder (TMD), Joint disease, Facial pain

## Abstract

**Aims:**

Temporomandibular disorder (TMD) is believed to be co-morbid with rheumatologic conditions such as Osteoarthritis (OA). We determine 30-day prevalence and cumulative incidence, and risk factors for facial pain in a cohort of subjects who either had or were at risk of developing symptomatic radiographic knee osteoarthritis (SRKOA).

**Methods:**

Poisson regression models examined whether age, sex, race, Center for Epidemiologic Studies-Depression Scale (CES-D) score, number of painful joints, and presence of SRKOA were risk factors for facial pain in 4,423 subjects at baseline and in 3,472 subjects at 24 and/or 48 months follow-up.

**Results:**

At baseline, 30-day period prevalence of facial pain was 9.25%; and 30-day cumulative incidence at 24-months and at 48-months was 5.9% and 4.9%, respectively. Factors associated with prevalence and incidence of facial pain were: younger age, female sex, (CES-D) score, and a larger number of painful joints. For each increase in age of one year, the incidence of facial pain decreased by 1%. Women had a 96% higher incidence than men, and each unit increase of (CES-D) score was associated with a 2% increase in the incidence of facial pain. For every additional painful joint, there was a 21% increase in the incidence of facial pain. Subjects with SRKOA had a 33% increase in the incidence of facial pain compared to those with risk factors for SRKOA.

**Conclusion:**

OA and TMD share several risk factors. The risk factors identified in cross-sectional analysis of prevalence are similar to those identified in longitudinal analysis on incidence.

## Introduction

Temporomandibular disorder (TMD) is a group of conditions affecting the joints and muscles of the craniofacial complex. Estimates of the prevalence of TMD among US adults vary, ranging between 4.6 and 12% [1–4]. Risk factors for TMD have been investigated in the “Orofacial Pain: Prospective Evaluation and Risk Assessment” (OPPERA) project in the US [5]. TMD is associated with being younger, being female, and being white [1,2]. For example, white women are twice as likely to report facial pain compared to white men [5]. Moreover, TMD is also associated with depressive symptoms, anxiety, sleep disturbance, and rheumatologic conditions including fibromyalgia, rheumatoid arthritis, and osteoarthritis (OA) [6–15]. Arthritides are the most prevalent rheumatologic conditions, affecting approximately a quarter of the adult population in the US [16]. OA is the most common type of arthritis, with 27 million US adults estimated to have signs and symptoms of OA [16–18]. Estimates of the prevalence of OA vary based on the signs and symptoms used to define the condition, and also on the joint and the age group studied. In the US, the prevalence of radiographic OA, irrespective of symptoms, ranges between 14% and 37%, while the prevalence of symptomatic radiographic OA ranges between 5% and 17% [17]. OA is positively associated with age in all ethnic groups [17], which contrasts with findings that, in white adults, TMD is inversely associated with age. However, OA shares several commonalities with TMD, such as being more prevalent in females [1,17,19–21]. Although studies have demonstrated the co-existence of widespread body and joint pain in individuals with TMD [6,21,22], few studies have investigated whether the extent of joint pain (the number of painful joints) or having symptomatic radiographic OA are risk factors for TMD. The aims of this study were to determine the 30-day prevalence and cumulative incidence of, and the risk factors for, facial pain in a cohort of 45 to 79 years old subjects who either had or were at risk of developing symptomatic radiographic knee osteoarthritis (SRKOA).

## Methods

### Data source

The data used in this study were from the “Osteoarthritis Initiative (OAI): A Knee Health study”, a US national prospective cohort study that was designed to identify and validate biomarkers for the development and progression of SRKOA [23]. The OAI data are anonymized and are publically available (https://www.oai.ucsf.edu/). The datasets used were the baseline dataset (v 0.2.2), the 24-month clinical dataset (v 3.2.1), and the 48-month clinical dataset (v 6.2.2), when the facial pain questionnaire was administered.

### Subjects and subcohort definitions

Subjects aged 45 to 79 years were enrolled at four sites in the US [23]. At enrollment, the subjects were assigned to one of three subcohorts: (1) the “progression” subcohort, (2) the “incidence” subcohort, and (3) a control group of 122 subjects, which was intended to determine the biological baselines within the OAI study (which was not used in this study). The names of the groups reported in this paper reflect the terms used by the OAI study at its inception. The progression subcohort was created to identify factors associated with the progression of disease in subjects who already had SRKOA at baseline. SRKOA was defined as the presence, within the previous 12 months, of frequent knee symptoms (aching, discomfort, pain, or stiffness on most days for at least one month), along with evidence of radiographic tibiofemoral knee OA in the symptomatic knee. The incidence subcohort was created to identify risk factors for the development of SRKOA (in those with a lack of radiographic evidence of the disease at baseline).The eligibility criteria for the incidence subcohort included having (1) knee pain within the previous 12 months *without* radiographic evidence of tibiofemoral OA in the painful knee or (2) other risk factors for SRKOA. The risk factors for SRKOA included being overweight, having a history of knee injury or prior knee surgery, frequent knee bending, a family history of total joint replacement, or having OA of the hand. For a full list of the exclusion criteria, see the protocol on the OAI website (https://www.oai.ucsf.edu/).

### Study variables

Demographic data (including data on age, sex, and race) were collected over the phone during the initial eligibility interviews. Once a participant was deemed to be eligible for screening, they were scheduled for a clinic visit to confirm their eligibility, obtain informed consent, and to collect data (by interview) regarding symptoms of arthritis and pain (including symptoms of facial pain).

### Temporomandibular joint (TMJ) symptoms

Facial pain characteristic of TMD was used to indicate the presence or absence of TMD. Self-reported facial pain, which can be a symptom of TMD, has previously been used in the US National Health Interview Surveys (NHISs) to estimate the prevalence of TMD [24]. This study analyzed data obtained from two questions about facial pain symptoms, which have been shown to be reliable and valid for measuring self-reported facial pain [25,26]. The presence, or absence, of facial pain characteristic of TMD was determined by the subjects’ answers to questions asked by study personnel during the screening visit and every two years during follow-up visits. The original OAI protocol included a total of 12 questions related to TMD that encompassed symptoms. We used answers from two of the questions (Table 1) to define the 30-day period prevalence and 30-day cumulative incidence of facial pain. Using 30-day recall periods to measure self-reported pain symptoms has been shown to be reliable by several studies [27–29]. The potential answers to the questions on facial pain were *yes, no, and don’t know/unknown/uncertain*. We grouped answers of *don’t know/unknown/uncertain* with *no*. If the participants answered *yes* to either one of the questions, they were deemed to have facial pain. If the participant answered *no* or *don’t know/unknown/uncertain* to both questions, the subject was considered to be free of facial pain.

### Knee, hip, and back pain

During the OAI screening visit and the follow-up visits, participants were asked five questions about pain in their knees, hips, and back (Table 1). We used the subjects’ responses to these five questions on bodily pain to determine the extent of pain that could reasonably be associated with joint-related pathology. The potential answers to the questions were *yes, no, don’t know, and refused*. As for the facial pain questions, *don’t know* and *refused* where recoded to *no*. An ordinal pain variable (ranging from zero to five) was created by summing the number of times the subject responded *yes* to the five joint pain questions.

### Depressive symptoms

At enrollment and at each of the follow-up visits, the Center for Epidemiologic Studies Depression Scale (CES-D) was used to identify depressive symptoms [30]. The 20-item CES-D explores depressed mood, feelings of guilt and worthlessness, feelings of helplessness and hopelessness, psychomotor retardation, loss of appetite, and sleep disturbance. The answers to most of the questions were scored as 0 *(rarely or none of the time)*, 1 *(some or a little of the time)*, 2 *(occasionally or more moderate amount of the time), or* 3 *(most or all of the time)*. The final score (which had a maximum value of 60) was obtained by summing the individual question scores, with the exception of inverting the scores of four out of the twenty items.

### Statistical analysis

Univariate descriptive analyses (which included calculating frequencies and percentages for the categorical variables on race and subcohort; medians and interquantile ranges for the categorical variable on the number of painful joints; and means and standard errors for the continuous variables on age and CES-D score) were used to determine the baseline characteristics, by sex, in the study sample. Poisson regression was used to model (1) the 30-day prevalent facial pain at baseline, and (2) the 30-day cumulative incidence of pain in subjects who had not previously reported pain. For the incidence analysis, a subject was considered to have developed facial pain if they had been free of facial pain at baseline but reported pain at 24 months, or if they were pain free both at baseline and 24 months but reported pain at 48 months. As subjects in the incidence analysis could contribute data at three time-points (baseline, 24 months, and 48 months), we used a generalized estimation equation (GEE) [31] to account for the correlation of serial observations from the same subject. The models included six independent variables (age, sex, race, CES-D score, number of painful joints, and subcohort (progression or incidence subcohort). The six variables were chosen based on existing knowledge about risk factors for facial pain. The incidence analysis also included period (baseline, 24 months, or 48 months). The analysis of the prevalence of facial pain at baseline included 4,423 subjects who had complete data on facial pain and each of the six independent variables. The incidence analysis included subjects who were free of facial pain both at baseline and at their prior OAI visit, if the data related to the 48-month visit. If a subject developed facial pain at 24 months, he or she was included as an incidence case of TMD at 24 months and was then dropped from the analysis (i.e. the associated 48-month follow-up data were not used in the analysis). If the subject was free of facial pain at 24 months, his or her 48-month follow-up data was used in the analysis. All analyses were performed with SAS 9.2 and 9.3.

## Results

### Subjects

**Figure 1:**
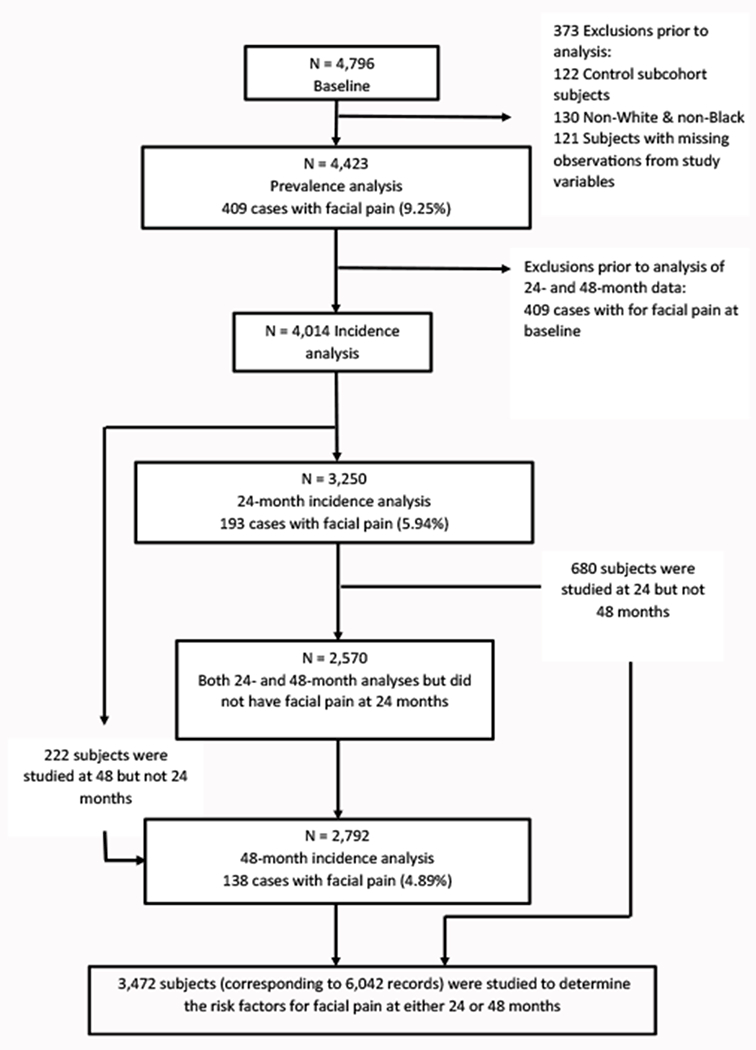
OAI study sample from baseline to the 48-month follow-up.

The OAI study included 4,796 subjects. We excluded the 122 subjects who had no evidence of, of risk factors for, SRKOA that were enrolled in the control subcohort (Figure 1). An additional 130 subjects who identified their race as something other than white or black and 121 subjects who had missing data on facial pain or any of the six independent variables were excluded. Several subjects had more than one reason for being excluded. In total, 4,423 were included in our analysis of the prevalence of facial pain at baseline. Of these 4,423 subjects, 409 (9.25%) had facial pain. These 409 subjects were excluded from the analyses of the incidence of facial pain, leaving 4,014 subjects who were eligible to contribute follow-up data. Twenty-four months after baseline, 3,250 of the 4,014 subjects who were studied at baseline were available for study. Forty-eight months after baseline, 2,951 of the 4,014 subjects who were studied at baseline were available for study. After dropping 159 subjects who were identified as having facial pain at 24 months, 2,792 subjects were available for study at 48 months. A total of 3,472 subjects were followed to either 24 or 48 months or both 24 and 48 months, contributing 6,042 subject records to the analysis of the risk factors for the incidence of facial pain. Of the 3,472 subjects, 2,570 subjects were studied at both 24 and 48 months, 680 were studied at 24 but not 48 months, and 222 were studied at 48 but not 24 months.

### Baseline characteristics of subjects followed for the incidence analyses

At baseline, the mean age (±standard error) of the 4,014 subjects included in the analysis of risk factors for the incidence of facial pain was 61.6±0.14 years; 44% of the subjects were men and 56% were women; 82% were white and 18% were black; and 29% were members of the progression subcohort and 71% were members of the incidence subcohort (Table 2). Although there were differences between men and women regarding the mean age, the mean CES-D score, and the mean number of painful joints, the differences were small and of minor clinical importance.

### Prevalence of facial pain at baseline

Among the 4,423 subjects at baseline, 409 reported facial pain, leading to a 30-day prevalence of 9.25% (95% CI: 8.43 to 10.14). The multivariate Poisson regression model that used data on the 30-day prevalence of facial pain (Table 3) showed that there was an inverse linear relationship between age and facial pain. The prevalence decreased by 2% for each additional year of age (prevalence: 0.98/year of age, 95% CI: 0.97 to 0.99; 0.79/decade of age). Women had a higher rate of facial pain compared to men (prevalence 2.39, 95% CI: 1.90 to 3.00). White people had a 33% higher rate of reporting facial pain compared to black people (prevalence 1.33%, 95% CI: 1.06 to 1.67). For each unit increase in CES-D, the rate of reporting facial pain increased by 3% (prevalence 1.03, 95% CI: 1.02 to 1.04). For each increase in the number of painful joints, the subjects had a 36% higher report of facial pain (prevalence 1.36/joint, 95% CI: 1.27 to 1.45). Subjects in the incidence subcohort had a 37% higher rate of facial pain compared to subjects in the progression subcohort (prevalence 1.37, 95% CI: 1.12 to 1.69). A model that included an interaction term between the number of painful joints and the subcohort showed no evidence of an interaction (p<0.97). In an analysis that was limited to data on the 2,574 women at baseline, there was evidence of an interaction between age and race (p<0.051). The prevalence was essentially unchanged with age in black women (prevalence 1.003/year of age), and decreased by 3%/year of age in white women (prevalence 0.97/year of age).

### Risk factors for developing facial pain at 24 and 48 months

Among the 3,250 subjects studied at 24 months (none of whom had facial pain at baseline), 193 developed facial pain, which corresponded to a 30-day cumulative incidence of 5.94% (95% CI: 5.18 to 6.81). Among the 2,792 subjects followed to 48-months (none of whom had facial pain at baseline or at 24 smonths), 138 developed facial pain, which corresponded to a 30-day cumulative incidence of 4.94% (95% CI: 4.2 to 5.81). The cumulative incidences were not significantly different (Pearson’s χ^2^: p<0.11). Among the subjects 3,472 subjects followed to 24 or 48 months, a total of 331 developed facial pains. The Poisson regression used to model incident cases of facial pain at 24 and 48 months (Table 3) demonstrated that the incidence decreased by 1% for increase in age of one year (incidence 0.99/year of age, 95% CI: 0.97 to 1. 00, or 0.87/decade of age). Women had a 95% higher report of facial pain than men (incidence 1.96, 95% CI: 1.54 to 2.48). White people had a 17% higher rate of reporting facial pain than black people (incidence 1.17, 95% CI: 0.88 to 1.55). For each unit increase in CES-D, the rate of reporting facial pain increased by 2% (incidence 1.02, 95% CI: 1.01 to 1.03). For each unit increase in the number of painful joints, subjects had a 21% higher report of facial pain (incidence 1.21/joint, 95% CI: 1.12 to 1.30). The incidence subcohort had a 33% higher rate of facial pain than the progression subcohort (incidence 1.33, 95% CI: 1.05 to 1.70). A model that included an interaction term between the number of painful joints and the subcohort indicated no evidence of an interaction (p<0.19). In an analysis that was limited to the 1,929 women who were followed to assess the incidence of TMD, there was no evidence of an interaction between race and age (p<0.98).

### Loss to follow-up

Although the results of the cross-sectional analysis (using baseline data on the prevalence of facial pain) and longitudinal analysis (on the incidence of facial pain) were similar, small differences in the magnitude of the odds ratios were observed between the analyses. The differences are likely to have been due to differential loss to followup. The subjects who were followed to 24 or 48 months compared to those seen at baseline were younger, had slightly lower CES-D scores, had fractionally fewer painful joints, were more likely to be in the incidence subcohort, were more likely to be men (p<0.08), and were more likely to be white than black (Table 4).

## Discussion

While others have looked at risk factors for TMD using prospective studies [32,33], we are unaware of any other studies that have examined the relationship between the severity of OA (i.e. the number of painful joints and the presence of symptomatic radiographic OA) and facial pain. In addition, our paper compares the results obtained from a cross-sectional analysis of risk factors for facial pain and the results obtained from a longitudinal analysis.

At baseline, all six independent variables were significantly associated with the number of prevalent cases of facial pain (Table 3). All of the risk factors except for race were significant predictors of the number of incidence cases of facial pain at the 24- and 48-month follow-ups. Although the adjusted estimates of some of the coefficients for the longitudinal analysis were attenuated compared to those in the cross-sectional analysis, they were in the same direction (i.e. they had the same sign). The only significant difference was for the variable on the number of painful joints, where the difference in prevalence at baseline and incidence at 24 and 48 months follow-up was statistically significant (p<0.027). Both cross-sectional analyses (on the prevalence of conditions) and longitudinal analyses (on the incidence of conditions) are subject to bias [34]. When the findings from a cross-sectional analysis are similar to those from a longitudinal analysis, as in this study, the likelihood of significant bias is decreased. Bias may have decreased the estimates associated with the incidence of TMD towards the null, but it did not change any of the risk factors to protective factors. This suggests that cross-sectional analyses can be used to derive valid inferences about TMD. The OPPERA project is a prospective cohort study that aimed to explore risk factors that were believed to be associated with TMD. The baseline case-control study arm showed that among the 200 cases that had TMD-like symptoms, there was a prevalence of examiner-verified TMD of 10.5%. Despite this finding being confirmed by a clinical Research Diagnostic Criteria for Temporomandibular Disorders (RDC/TMD) examination, it closely approximates our findings of a prevalence of self-reported facial pain of 9.25%. The OPPERA incidence analysis found that, out of 2,737 subjects who were followed up for 5.2 years, the incidence of examiner-verified TMD was 4%, while our study estimated a 30-day cumulative incident of 5.94% and 4.89% at 24- and 48-months respectively [35]. Although there are fundamental differences in the study design between the OPPERA and the OAI, the results from the OPPERA project confirm that findings from self-reports can be as valid as those associated with clinical examinations. A unique aspect of our study is that we found that having an increasing number of painful joints increased the risk of developing facial pain in both the cross-sectional and the longitudinal analyses. This suggests that OA and TMD may share pathophysiologic elements. We are only aware of one paper that quantified the prevalence of jaw pain in subjects with OA [36]. Wolfe et al. analyzed data from the National Data Bank for Rheumatic Diseases (NDB), which included rheumatologist clinics’ sample of 22,720 participants, 4,011 of whom had been diagnosed with OA [36]. The evaluation of jaw pain was carried out using a single item of the Regional Pain Scale. The study reported a prevalence of jaw pain of 18.6% [36]. Therefore, the prevalence and incidence of facial pain among a cohort who were at high risk of developing TMD (as a result of having SRKOA or risk factors for SRKOA) has not, to our knowledge, been previously reported. In addition, much of our knowledge regarding TMD comes from studies of younger subjects. The OAI study included middle-age and older subjects and a large number of both black and white people, which allows both a wide age range and the effects of race to be explored. We found that the incidence of facial pain decreased with increasing age. Data from the US National Health Interview Survey (NHIS) from 2002–2005 and 2007–2009 demonstrated an inverse linear relationship between age and self-reported facial pain in non-Hispanic white women [1,5,20]. We found that women were at higher risk of developing facial pain than men, with a ratio of 2:1. Similar results have been reported by others [1–3,5,11,37,38]. The relationship between age, race, and TMD may be complex. The 2000–2005 NHIS data revealed that the highest prevalence of TMD pain was in White women who were in their childbearing years, with the prevalence declining post-menopause [1,3,5,12,20]. In contrast, Self-reported TMD pain amongst black women either remained unchanged or increased in post-menopause. Our results from the baseline data, but not from the follow-up data, support the previous findings of different age slopes in white women compared to in black women.

Our findings that the 30-day prevalence of self-reported facial pain at baseline was 9.25% is similar to values reported in other studies. For example, Riley et al. reported a six-month period prevalence of 8.3% for jaw joint pain and 3.1% for facial pain among men and women [3]. In addition, Janal et al. [39] conducted a telephone survey of women in New York using the same question on jaw joint pain that was used in this study. The authors reported a six-month period prevalence of 10.1%.The estimate dropped to 4.4% when those who experienced headaches and pains lasting less than two weeks in the same anatomical area were excluded. Despite reports of low sensitivity (42.7%) for the jaw joint pain question, results analyzed from our study, the Janal et al. [39] and the OPPERA baseline case control study were all similar. While the NHIS 2000–2005 data indicated a three-month prevalence of 4.6% for self-reported TMJ and muscle disorders pain among US adults [1,21], half the estimate of this study’s prevalence, it is possible that recalling pain over a three-month is not as accurate as recall over a 30-day period [28], or that the prevalence of TMD-like pain among patients with OA is higher than that among the US adult population. Individuals with TMD pain often report pain in adjacent areas, such as the neck and shoulders and the back, as well as in other areas of the body [6,21,40,41]. Based on our hypothesis that the TMJ may represent an additional OA-affected joint, we examined the relationship between the number of painful joints and facial pain. We found a positive linear relationship between the number of painful joints and the prevalence and incidence of facial pain. Subjects in the incidence subcohort had a higher risk of reporting facial pain compared to those in the progression subcohort. Investigating the reasons behind this differential presentation would require a more extensive analysis, which is beyond the scope of this paper. Our findings should be interpreted with the caveat that the self-reported facial pain was not validated by clinical examinations, and thus positive responses could have reflected pain due to other conditions affecting the regions of interest, such as toothache, earache, and headache. Studies carried out on adolescents have showed that self-reported pain in the TMJ region [32], whether consistent with a diagnosis of TMD or not, is reported more frequently by females compared to males, a finding that often also applies in adults. This may partially explain our finding of a higher risk for women than men. Although self-reported facial pain is not exclusive to TMD, it is one of the most commonly reported symptoms, and much of the epidemiological literature on pain, including the literature on TMD, uses self-reported facial pain as a surrogate for clinical examinations [26,42]. Moreover, self-reported facial pain constitutes an integral part of the case history element of the questionnaire associated with Axis I of the RDC/TMD (currently renamed to the Diagnostic Criteria for Temporomandibular Disorders [DC/TMD]) examination [26,43]. One limitation of the study is that it is based on data on pain symptoms from two 30-day recall periods (at 24 and 48 months after the start of the study). It is important to note that most longitudinal studies do not collect data on pain using a recall period of one day. They generally use either a three-month recall period (which was used in the NHIS studies [1,21]) or a 30-day recall period at three-month follow-up visits (as was done in the OPPERA study [5]). The literature indicates that pain severity is related to the accuracy of pain recall [29]. Severe pain generally indicates the presence of a more severe disease and warrants further clinical investigations. Therefore, over the 48 months of follow-up, it is likely that those who had clinically significant facial pain were the patients who recalled their pain experiences at the follow-up visits. An additional limitation of the study is the inconsistency in the recall period associated with the questions on pain other than TMJ pain. The hip pain question asked about pain over the previous 12 months, as opposed to the 30-day recall periods associated with the other joint pain questions (the TMJ, left knee, right knee, and back pain questions). Despite the differences in the recall periods, our analyses showed that an increasing number of painful joints were associated with a higher prevalence and a higher incidence of facial pain. We are, to our knowledge, the first investigators to report the prevalence of, and the incidence of, self-reported facial pain from a sample of subjects who either had, or were at a high risk of developing, OA. An advantage of studying risk factors for TMD in the subjects that participated in the OAI study is that the OAI subjects were likely to have been at increased risk of developing TMD. Facial pain, the outcome explored by this study, was assessed by reliable and valid questions that have been used in prior studies. An important aspect of our study is that we used data from a population with a wider and older age range than that which is usually included in studies of TMD. These data are therefore more relevant to clinicians, especially those who treat patients with polyarticular OA. In conclusion, this study demonstrates that the major risk factors for developing facial pain in a cohort of middle-aged and older subjects are being female, having depressive symptoms, and having a higher number of painful joints. These factors overlap with the risk factors for developing OA. However, unlike for OA, black people and older people tend to be less likely to self-report facial pain.

## Figures and Tables

**Table 1:** OAI questionnaire items.

Joint	Location	Question
TMJ	Joint	“During the past 30 days have you experienced pain or aching in your jaw joint or in front of your ear?”
	Face	“During the past 30 days have you experienced pain or aching across your face or cheek?”
Knee	Right	“During the past 30 days, have you had any pain, aching, or stiffness in your right knee?”
	Left	“During the past 30 days, have you had any pain, aching, or stiffness in your left knee?”
Hip	Right	“During the past 12 months, have you had any pain, aching or stiffness in or around your right hip?”
	Left	“During the past 12 months, have you had any pain, aching or stiffness in or around your left hip?”
Back	Any	“During the past 30 days, have you had any back pain?”

OAI: Osteoarthritis Initiative; TMJ: temporomandibular joint

**Table 2:** Characteristics of subjects at baseline (without symptoms of TMD) that were followed for 24 or 48 months to determine the risk factors for incident cases of facial pain, by sex.

Variable				Total				Men				Women				Men vs.
				n = 4,014				n = 1,762 (44%)				n = 2,252 (56%)				Women
				**Mean**		**SE**		**Mean**		**SE**		**Mean**		**SE**		**P value**‡
Age (years)				61.6		0.14		61.2		0.23		62.0		0.19		0.02
CES-D score				6.3		0.10		5.9		0.15		6.6		0.14		0.0005
				**Median**		**IQR**		**Median**		**IQR**		**Median**		**IQR**		**P value**†
Painful joints (n)†				1		1 to 2		1		1 to 2		1		1 to 2		0.0007
				**N**		**%**		**N**		**%**		**N**		**%**		**P value**¶
Race		White		3,274		82		1,517		86		1,757		78		0.0001
		Black		740		18		245		14		495		22		
Subcohort		Progression		1,182		29		541		31		641		28		0.13
		Incidence		2,832		71		1,221		69		1,611		72		

CES-D, Center for Epidemiologic Studies - Depression Scale; IQR, inter-quartile range; SE, standard error; TMD, temporomandibular joint disorder

‡ Student’s t-test

† Wilcoxon rank sum test

¶ Pearson’s χ^2^ test.

**Table 3:** Poisson regression predicting number of prevalent cases of TMD at baseline and the incidence at follow-up.

Independent Variable	Baseline								
	(N=4,423 subjects, 409 with facial pain)								
	Coefficient*	SE	P value	**Prevalence**†	95% CI				
Intercept	−2.825	0.393	0.000	0.06	0.03	to	0.13		
Age (years)	−0.024	0.006	0.000	0.98	0.97	to	0.99		
Sex (female vs. male)	0.871	0.122	0.000	2.39	1.88	to	3.04		
Race (White vs. Black)	0.286	0.128	0.025	1.33	1.04	to	1.71		
CES-D score	0.029	0.005	0.000	1.03	1.02	to	1.04		
Painful joints (n)	0.307	0.037	0.000	1.36	1.27	to	1.46		
Subcohort (incidence vs. progression)	0.318	0.115	0.006	1.37	1.10	to	1.72		
	**Follow-up (at 24 and 48 months)**								**Baseline vs.**
	(N=3,472 subjects, 331 with facial pain)								**Follow-up**
	**Coefficient**‡	**SE**	**P value**	**Incidence**¶	**95% CI**				**P value**
Intercept	−3.187	0.4353	0.000	0.04	0.02	to	0.10		0.536
Age (years)	−0.015	0.0063	0.022	0.99	0.97	to	1.00		0.289
Sex (female vs. male)	0.673	0.1219	0.000	1.96	1.54	to	2.49		0.251
Race (White vs. Black)	0.157	0.145	0.280	1.17	0.88	to	1.55		0.504
CES-D score	0.019	0.0064	0.004	1.02	1.01	to	1.03		0.210
Painful joints (n)	0.191	0.0378	0.000	1.21	1.12	to	1.30		0.027
Subcohort (incidence vs. progression)	0.288	0.1236	0.020	1.33	1.05	to	1.70		0.858

CES-D: Center for Epidemiologic Studies - Depression Scale; CI: confidence interval; SE: standard error; TMD: temporomandibular joint disorder

† Predicted number of prevalent cases of TMD at baseline for a unit change in the independent variable.

‡ The Poisson regression used a generalized estimation equation (GEE) to account for repeated observations (at 24 and 48 months) from the same subject. The analysis only included subjects who did not have TMD at baseline. Subjects without TMD at 24 months were followed to 48 months (as those who developed TMD at 24 months were considered to be cases and therefore were dropped from the analysis).

¶ Predicted number of incident cases of TMD (the sum of the number of new cases at 24 and 48 months) for a unit change in the independent variable.

**Table 4:** Comparison of the characteristics of subjects who were followed beyond baseline compared to those who were not followed.

Followed beyond baseline	Number of subjects	Age (years)		CES-D score		Painful joints (n)		
	N	Mean	SE	Mean	SE	Mean	SE	
No	542	65.5	0.46	7.5	0.32	1.6	0.06	
Yes	3472	61.0	0.15	6.13	0.11	1.5	0.02	
Difference	4014	4.45	0.42	1.40	0.31	0.13	0.06	
P value			0.000		0.000		0.0281	
	Subcohort				Sex			
	Progression		Incidence		Male		Female	
	**N**	**Row %**	**N**	**Row %**	**N**	**Row %**	**N**	**Row %**
No	183	15.5	359	12.7	219	12.4	323	14.3
Yes	999	84.5	2473	87.3	1543	87.6	1929	85.7
Total	1182	29.4	2832	70.6	1762	43.9	2252	56.1
P value				0.02				0.08

CES-D, Center for Epidemiologic Studies - Depression Scale; SE, standard error
